# Metal-*Pseudomonas veronii* 2E Interactions as Strategies for Innovative Process Developments in Environmental Biotechnology

**DOI:** 10.3389/fmicb.2021.622600

**Published:** 2021-03-03

**Authors:** María Pia Busnelli, Irene C. Lazzarini Behrmann, Maria Laura Ferreira, Roberto J. Candal, Silvana A. Ramirez, Diana L. Vullo

**Affiliations:** ^1^Área Química, Instituto de Ciencias, Universidad Nacional de General Sarmiento, Los Polvorines, Argentina; ^2^Consejo Nacional de Investigaciones Científicas y Técnicas (CONICET), Buenos Aires, Argentina; ^3^Instituto de Investigación e Ingeniería Ambiental (IIIA), Universidad Nacional de General San Martin, San Martín, Argentina

**Keywords:** biosensor, exopolymeric substances, metal removal and recovery, Cu nanoparticles, biosurfactant, *Pseudomonas veronii*

## Abstract

The increase of industrial discharges is the first cause of the contamination of water bodies. The bacterial survival strategies contribute to the equilibrium restoration of ecosystems being useful tools for the development of innovative environmental biotechnologies. The aim of this work was to study the Cu(II) and Cd(II) biosensing, removal and recovery, mediated by whole cells, exopolymeric substances (EPS) and biosurfactants of the indigenous and non-pathogenic *Pseudomonas veronii* 2E to be applied in the development of wastewater biotreatments. An electrochemical biosensor was developed using *P. veronii* 2E biosorption mechanism mediated by the cell surface associated to bound exopolymeric substances. A Carbon Paste Electrode modified with *P. veronii* 2E (CPEM) was built using mineral oil, pre-washed graphite power and 24 h-dried cells. For Cd(II) quantification the CPEM was immersed in Cd(II) (1–25 μM), detected by Square Wave Voltammetry. A similar procedure was used for 1–50 μM Cu(II). Regarding Cd(II), removal mediated by immobilized EPS was tested in a 50 ml bioreactor with 0.13 mM Cd(II), pH 7.5. A 54% metal retention by EPS was achieved after 7 h of continuous operation, while a 40% was removed by a control resin. In addition, surfactants produced by *P. veronii* 2E were studied for recovery of Cd(II) adsorbed on diatomite, obtaining a 36% desorption efficiency at pH 6.5. Cu(II) adsorption from a 1 mM solution was tested using *P. veronii* 2E purified soluble EPS in 50 mL- batch reactors (pH = 5.5, 32°C). An 80% of the initial Cu(II) was retained using 1.04 g immobilized EPS. Focusing on metal recovery, Cu nanoparticles (NPs) biosynthesis by *P. veronii* 2E was carried out in Cu(II)-PYG Broth at 25°C for 5 days. Extracellular CuNPs were characterized by UV-Vis spectral analysis while both extracellular and intracellular NPs were analyzed by SEM and TEM techniques. Responses of *P. veronii* 2E and its products as biosurfactants, bound and soluble EPS allowed Cu(II) and Cd(II) removal, recovery and biosensing resulting in a multiple and versatile tool for sustainable wastewater biotreatments.

## Introduction

The increase of industrial activities together with the consequent effluent discharges, is the first cause of the contamination of water bodies from anthropogenic origin. The generation of high hazardous pollutants compromises water quality with the consequent severe environmental damages including human health (Dai et al., 2020).

In this context, environmental relevance metals (ERM) are considered as potentially toxic pollutants even at small concentrations as are usually involved in the bioaccumulation process through the food chain. The non-biodegradable nature of these metals and their accumulation cause detrimental effects to the wide diversity of life forms (Leong and Chang, 2020).

Several techniques have been employed for the removal or recovery of ERM from polluted ecosystems. Some of the known established remediation processes include membrane filtration and chemical precipitation, redox reactions, ion-exchange, reverse osmosis, etc. (Kiran Marella et al., 2020). However, these conventional methods are often found to be unsustainable practices from the environmental perspective and cannot be implemented at higher scale due to their cost. Hence, biological treatments could be a suitable alternative and comparably inexpensive to conventional remediation strategies for the removal of the ERM from contaminated habitats (Mahapatra et al., 2020).

The diversity of survival strategies in bacteria contributes to the equilibrium restoration of damaged ecosystems and constitutes a group of useful tools to be exploited in the development of innovative environmental biotechnologies. Bioremediation techniques involving microorganisms have driven researchers’ attention due to their highly effective eco-friendly nature. Toxic metals in active species can affect the microbial population dynamics and induce indigenous microbial responses to such stress. One of such adaptations is the ability to produce extracellular polymeric substances (EPS) as a protective barrier which avoids the ERM uptake. The interaction of metals with the EPS matrix usually occurs through an adsorption process as consequence of the presence of negatively charged functional groups (Parmar et al., 2020).

The bioremediation mediated by EPS as biosorbents is one of the most promising processes from an economically viable point of view. EPS are products of microbial secretions, typically comprising polysaccharides, and proteins. These biopolymers have functional groups such as carboxyl, hydroxyl, phosphate, and amine groups, which enables them to retain ERM (Ajao et al., 2020; Cao et al., 2020). Due to the ability of metal sorption, EPS can mitigate toxicity toward microorganisms by coordination reactions between metals and EPS ligands at low metal concentrations (mg L^–1^ range), even preventing their diffusion into the deeper parts of biofilms (Sudmalis et al., 2020). The main characteristic of EPS is to enhance the aggregation of bacterial cells. Adhesion and cohesion occur between EPS and biomass leading to the formation of flocs. As a consequence, EPS are widely used for many applications such as sludge flocculation, settling, dewatering, metal binding, and removal of toxic organic compounds (Nouha et al., 2018; Shen et al., 2018).

Chemical analysis of such environmental samples requires transportation from site to the laboratory in order to apply useful analytical methods such as atomic absorption spectroscopy, electron capture devices, inductively coupled plasma optical emission, mass spectrometry, etc. The aforementioned methods involve the use of expensive equipment, are time-consuming and require trained personnel. The fact that these technologies may not be accessible worldwide, particularly in developing countries, points to the need of developing simpler and easy to handle devices to access affordable monitoring without the requirement of trained personnel. According to the International Union of Pure and Applied Chemistry (IUPAC), a biosensor can be defined as “a device that uses specific biochemical reactions mediated by isolated enzymes, immunosystems, tissues, organelles or whole cells to detect chemical compounds usually by electrical, thermal, or optical signals” (Nagel et al., 1992). In this way, biosensors can be applied in environmental monitoring and offer several advantages such as specificity, sensitivity, portability, and miniaturization. They require minimal sample preparation (Rodriguez-Mozaz et al., 2006; Palchetti and Mascini, 2008) and allow rapid and reliable detection of the target substance (Saleem, 2013). Basically, in a biosensor occurs the recognition of a target by a biological sensing element and this event is turned into a measurable signal. The microbial machine, isolated components and excreted products have been applied to the detection of analytes in fields such as diagnostic, environmental monitoring, food, and safety (Ejeian et al., 2018; Nosrati et al., 2018; Griesche and Baeumner, 2020; Guo et al., 2020). Whole cells, biomolecules such as enzymes and DNA, metabolites such as EPS, tensioactives, and siderophores have been used as biological sensing elements (Lei et al., 2006; Park et al., 2013; Hussain et al., 2017; Kim et al., 2017; Ferapontova, 2018; Guo et al., 2020; Kucherenko et al., 2020). Combination of these sensing elements with electrochemical methods make up an interesting alternative to produce affordable *in situ* monitoring with adequate sensitivity (Lu et al., 2018; Kucherenko et al., 2020).

Carbon paste electrodes (CPE) are built using a homogenous mixture of carbon power and non-conductive binding. They are inexpensive, easy to miniaturize and modify, allowing new and reproducible surfaces with a wide operating potential window (Aglan et al., 2019; Laghrib et al., 2019). CPE modified with nanoparticles, nanotubes, whole cells, enzymes, etc., have shown an increase in sensitivity and selectivity (Devnani et al., 2014). CPE have been modified with bacterial cells (Yüce et al., 2011; Mohanraj et al., 2020), yeast (Yüce et al., 2010; Akyilmaz et al., 2017), fungus (Alpat et al., 2008), and plant components (Asheri et al., 2019; Jackfama et al., 2019) and only a few are applied in environmental metal detection. A Pb(II)-microbial biosensor used *Pseudomonas aeruginosa* heat-dried biomass (Yüce et al., 2011). Recently, the use of live *P. aeruginosa* cell suspensions in combination with drop coating of the electrode has been reported regarding surface modification (Mohanraj et al., 2020). For Cu(II) detection, a *Circinella* sp. biosorption-based biosensor was developed with a lineal range from 0.5 to 10 μM (Alpat et al., 2008) and a *Rhodotorula mucilaginosa* modified microbial sensor with a lineal range from 0.1 to 10 μM (Yüce et al., 2010). For Cd(II) detection a *Typha latifolia* root-modified CPE was characterized (Martínez-Sánchez et al., 2012). Devnani et al. (2014) reported a black rice extract-CPE for Pb(II), Cd(II), Cu(II), and Zn(II) detection.

An important area of research in nanoscience is related to the synthesis of metallic nanoparticles (NPs). These particles show great diversity with many applications in medical and industrial areas. The most common methods for preparing nanoparticles are physicochemical, which generally use toxic reagents with complex and expensive synthesis steps. Some processes involve the application of surfactants, solvents, elevated temperatures, and pressures, with generation of hazardous by-products and limiting the fields where they can be applied (Dauthal and Mukhopadhyay, 2013; Hussain et al., 2016). The use of microorganisms for extracellular or intracellular NPs biosynthesis is emerging as an alternative to chemical methods in agreement to a growing need to develop more economical, non-toxic, and environmentally sustainable synthesis methods. Some microorganisms, including bacteria, survive and are well adapted to environments with high metal concentrations as industrial effluents. As a consequence, microbes developed detoxification mechanisms, resulting in some cases in the formation of metallic NPs (Dissook et al., 2013), allowing recycle metals contained in wastewaters. Particularly bacteriogenic Cu-NPs have been characterized by their anticancer, antibacterial, and antifungal activities in addition to catalytic, optical, and electrical properties (Ghosh, 2018).

The aim of this work was to study the Cu(II) and Cd(II) biosensing, removal and recovery, mediated by whole cells, exopolymeric substances (EPS) and biosurfactants of the indigenous and non-pathogenic *Pseudomonas veronii* 2E, isolated from polluted environments, to be applied in the development of innovative wastewater biotreatments.

## Materials and Methods

### *P. veronii* 2E EPS Production

*Pseudomonas veronii* 2E is an autochthonous bacterium isolated from sediments associated to the highly polluted Reconquista River basin (Buenos Aires Metropolitan Area). This strain was identified by 500 bp 16S r- RNA gene sequencing (Vullo et al., 2008). Cells grown at 25°C up to late exponential phase in 1.2 L of M9 broth [per liter: potassium phosphate dibasic (K_2_HPO_4_) 6 g; potassium phosphate monobasic (KH_2_PO_4_) 3 g; ammonium chloride (NH_4_Cl) 1 g; sodium chloride (NaCl) 0.5 g; 2% yeast extract 2.50 mL; 1 M calcium chloride (CaCl_2_) 1 mL; 1 M magnesium sulfate (MgSO_4_) 2.2 mL supplemented with 2% glycerol] were separated by centrifugation at 7000 *g* during 15 min. The soluble EPS were precipitated from culture supernatants by adding 2.2 volume of ethanol 96% (v/v) and stored overnight at −20°C. Next, the precipitated EPS were separated by centrifugation during 20 min at 7000 *g*, 4°C, repeating this procedure three times after resuspending EPS in ultrapure water (18.2 MΩcm, Millipore) (Ferreira et al., 2017). EPS fraction was finally resuspended in a minimum volume of ultrapure water (18.2 MΩcm, Millipore) and dialysed through cellulose membrane (Sigma-Aldrich, MW > 12400 Da) during 48 h to remove low molecular weight species. Purified EPS were dried at 50°C up to constant weight.

### Cd(II) Removal Mediated by EPS

**FIGURE 1:**
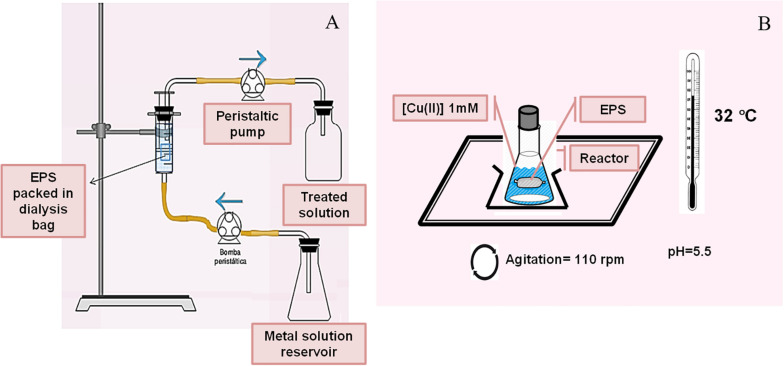
Continuous **(A)** and batch **(B)** reactors designed for metal biosorption by EPS.

Cd(II) removal from a model effluent was studied using EPS packed in pretreated dialysis bags. Commercial dialysis bags (cellulose membrane, Sigma-Aldrich, MW > 12,400 Da) were treated by boiling in 2% (m/v) NaHCO_3_, then in 1 mM EDTA and finally washed with ultrapure water (18.2 MΩcm, Millipore). The treated bag was filled with 12 mL of 3.3 mg mL^–1^ EPS solution, sealed and placed into a 65 mL maximum capacity syringe with a total volume of 50 mL 0.13 mM CdCl_2_ (10 mM HEPES – 2-[4-(2-hydroxyethyl)piperazin-1-yl]ethanesulfonic acid- pH 7.5). The metal solution was continuously circulated with a two-head peristaltic pump (Drive Watson Marlow 505S), from the bottom of the syringe (constant flow 9 mL h^–1^) to the top connected to another pump head, operating with a reactor volume of 50 mL (Figure 1A). Samples were taken from the upper outlet of the bioreactor at different times up to the Cd (II) concentration of the inlet was equal to the concentration of the outlet solution. To avoid the contribution of Cd(II) diffusion within the bag, a control experiment was carried out replacing the EPS solution by ultrapure water (18.2 MΩcm, Millipore). Cd(II) removal by a commercial ion exchange resin, Chelex^®^ 100 (Bio-Rad), was also explored using the same procedure.

Cd(II) quantification was performed by square wave voltammetry (SWV) combined with metal preconcentration at −0.75 V (vs. Ag/AgCl). Measurements were performed with an Autolab PGStat10 (Eco Chemie) and a Metrohm 663 VA polarographic stand (hanging mercury drop electrode mode). 30 s and 60 s were used as deposition time. A hanging mercury drop electrode N_2_ was bubbled through the solution for 120 s, after each metal addition.

### Copper Biosorption and Desorption Assays

In order to study the interaction between Cu(II) and the bacterial exopolymers, 1.04 g EPS obtained as previously mentioned were resuspended in 12 mL of ultrapure water (18.2 MΩcm, Millipore) in a dialysis bag and then located inside a batch reactor consisting in a 250 mL flask with 50 mL of 1 mM Cu(II) solution at pH 5.5 adjusted by 500 μL of 1 M MES (2-(N-morpholino)ethanesulfonic acid) buffer (Figure 1B). Metal biosorption kinetics was explored along 96 h at 25°C. Copper concentration in the supernatants was spectrophotometrically measured using the Bicinchoninic acid method (Benner and Harris, 1995). The metal adsorption capacity mediated by the EPS was expressed according to the equation 1.

(1) $$q=\frac{V\times \left(\mathrm{Ci}-\mathrm{Ceq}\right)}{m}$$

Where *Ci* is the initial Cu(II) concentration, *V* is the total volume of the biosorption mixture assayed and *m* is the total biosorbent mass as dry weight.

Once the biosorption assay was ended, the metal desorption was carried out using HCl. For that purpose, the batch reactor was purged and 17 mL of 75 mM HCl was added during 30 min under agitation at 32°C. Copper concentration in supernatant was quantified as already mentioned. The procedure was repeated up to three cycles. The metal desorption results were calculated according to equation 2.

(2) $$\%\mathrm{Cu}\left(\mathrm{II}\right)\mathrm{desorption}=\frac{\mathrm{Cu}\,\left(\mathrm{II}\right)\,\mathrm{mmol}\,\mathrm{desorbed}}{\mathrm{Cu}\,\left(\mathrm{II}\right)\,\mathrm{mmol}\,\mathrm{adsorbed}}\times 100$$

Biosorption and desorption experiments were performed by duplicates with the corresponding standard deviation calculation.

### Electrode Preparation for Cu(II) and Cd(II) Sensing

Carbon paste electrodes (CPE) were prepared by mixing acid pre-washed graphite powder from filler rod (Arcair^®^) (70%) and commercial mineral oil (30%) in a mortar. Bacterial biomass used to modify the CPE was obtained growing *Pseudomonas veronii* 2E in Nutrient Broth (Merck or OXOID) for 67–70 h (32°C, 120 rpm) up to stationary phase. After incubation, cells were harvested by centrifugation at 5432 *g* for 10 min and the pellet was washed twice with ultrapure water (18.2 MΩcm, Millipore). Finally, and prior to use, biomass was dried at 32°C for 24 h. The modified CPE (CPEM) was built using a homogenous paste of power graphite (60%), commercial mineral oil (30%), and *P. veronii* 2E dry biomass (10%). For both CPE and CPEM, the homogeneous mixture was firmly packed into a Teflon body and the electrode surface was smoothed on a weighing paper before use. CPEM and CPE were stored at room temperature until use.

Metal sensing can be described by a two-step procedure. The first one involved metal retention on the electrode surface and the second one consisted in the measurement step, which was accomplished by electrochemical techniques. CPE and CPEM were used in a three-electrode cell, provided with a Ag/AgCl reference electrode (KCl saturated, Metrohm) and a graphite bar as counter electrode. Nitrogen was used to eliminate oxygen between experiments. SWV combined with a preconcentration step was applied. Electrochemical experiments were controlled by a PGSTAT204 (Metrohm, Autolab) and software NOVA 1.10 (step potential 5 mV, amplitude 20 mV, frequency 25 Hz).

In the preconcentration step, metal retention was achieved by immersion of the CPEM in the solution containing the analyte. These solutions were prepared considering best biosorption conditions for *P. veronii* 2E (Vullo et al., 2008): for Cd(II) solutions 10 mM HEPES pH 7.5, 0.1 M NaNO_3_ and for Cu(II) 10 mM MES pH 5.5, 0.1 M NaNO_3_. Basically, the CPEM was exposed by immersion in 5 mL of the metal solution for 5 min without stirring. Retention was accomplished at room temperature for Cu(II) but in the case of Cd(II) the effect of temperature was explored and a thermostatic bath was required. Metal concentrations in the μM range were investigated [1–50 μM Cu(II), 1–25 μM Cd(II)].

After exposure to the metal solution, the CPEM was carefully washed with ultrapure water (18.2 MΩcm, Millipore), transferred to the electrochemical cell containing 0.1 M NaNO_3_ or 0.003 M HNO_3_ as supporting electrolyte for Cd(II) and Cu(II), respectively, and the current signal was recorded. Regarding this measurement step, different parameters affecting the current response such as deposition potential and deposition time were explored. CPEM surface cleanliness was achieved and controlled between experiments by immersion in a cleaning solution [30 s in 1.5 M HNO_3_ for Cu(II), 5 min in 0.5 M MES, pH 5.5 for Cd(II)]. At least three replicates for each cadmium or copper concentration were performed and standard errors were calculated.

Limit of detection (LOD) and limit of quantification (LOQ) were calculated as:

$$\mathrm{LOD}=\frac{3.\mathrm{Sa}}{m}$$

$$\mathrm{LOQ}=\frac{10.\mathrm{Sa}}{m}$$

where *Sa* is the standard error of the intercept from the regression line and *m* is the calibration curve’s slope (Madhuchandra et al., 2020).

### Biosynthesis and Characterization of Cu-NPs

Copper nanoparticles were biologically synthesized. *P. veronii* 2E was pre-cultured on the enriched medium PYG (g L^–1^: 5 peptone, 2.5 yeast extract, 1 glucose) at 20, 25, and 32°C, pH 5–6 and 120 rpm until late exponential phase of growth. A total of 10 mL culture was transferred into 100 mL of fresh medium supplemented with different CuSO_4_ concentrations (1.0, 1.5, or 2.0 mM) and incubated in the same conditions for 5 days. A non-inoculated medium was used as negative control. Supernatant was collected and centrifuged (7000 *g*, 10 min, 20°C), filtered through cellulose nitrate membrane to eliminate cells (0.45 μm filter) and stored to analyze extracellular Cu-NP biosynthesis. The pellet was washed twice with ultrapure water (18.2 MΩcm, Millipore) and stored for intracellular NP evaluation. Control [Cu(II) free-culture] was also run along with the experiments.

Cu-NPs were spectrophotometrically characterized combined with Scanning Electron Microscopy (SEM) and Transmission Electron Microscopy (TEM). Cell-free solutions were then investigated for extracellular Cu-NPs production by UV-Vis spectral analysis (400–800 nm) and compared with the controls. For Electron Microscopy analyses, a drop of the samples was seeded on silicon, dry at room temperature and subjected to SEM for NP observation (FE-SEM Zeiss SUPRA 40; CMA, FCEN-UBA). TEM was carried out to analyze intra-cellular Cu-NPs production. Samples were treated according to Venable and Coggeshall (1965) (Zeiss EM 109; CIME- INSIBIO-UNT-CONICET and UNSAM).

### Cd(II) Recovery From Diatomite Using Biosurfactants

**FIGURE 2:**
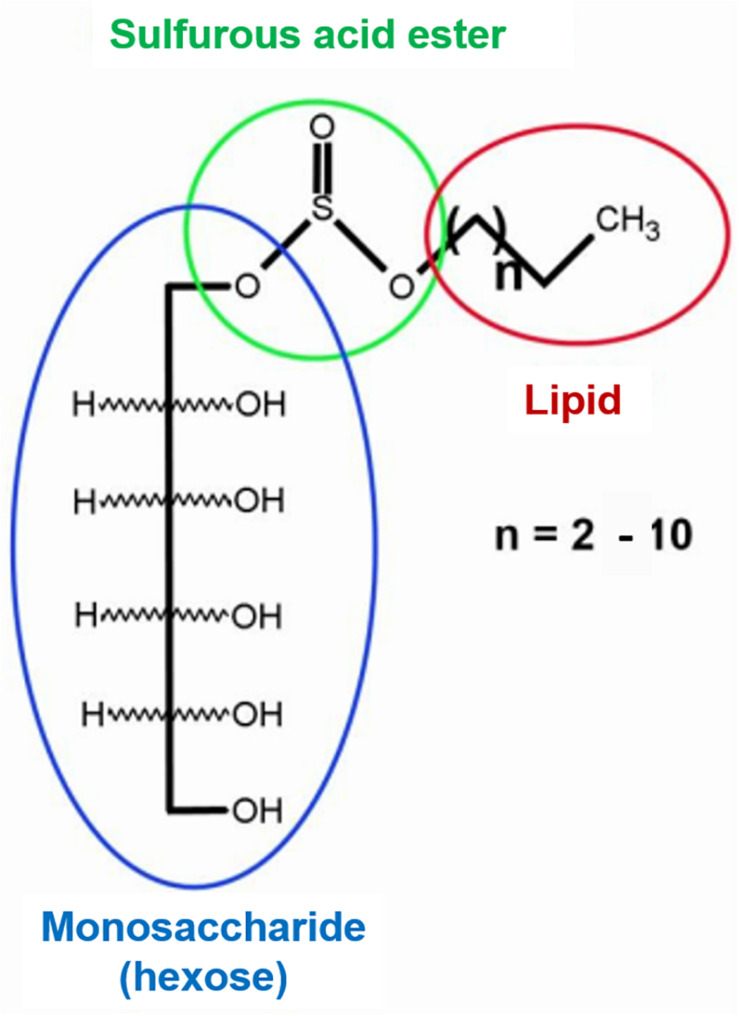
Chemical structure of secreted *Pseudomonas veronii* 2E biosurfactant.

Cd(II) recovery from diatomite was tested using *P. veronii* 2E biosurfactant (Figure 2; Barrionuevo, 2017). 5.0 g diatomite was exposed for 24 h to 500 mL 2.7 mM Cd(II) (pH 6.5, 25°C, 120 rpm). The resulting solid [Cd(II)-diatomite] was centrifuged (15 min, 3000 *g*), washed and dried up to constant weight (60°C). Under these conditions, 65.1% Cd(II) adsorption was obtained. For the recovery experiment, 0.5 g Cd(II)-diatomite were treated with 3 mL 0.5% (m/v) surfactant solution. *Pseudomonas aeruginosa* PA01 rhamnolipid solution was used as reference and ultrapure water (18.2 MΩcm, Millipore) as negative control. After a 48 h- incubation (25°C, 120 rpm) the suspension was centrifuged (15 min, 3000 *g*). The supernatant was collected and photooxidized during 24 h to eliminate organic matter for Cd(II) determination. In all cases Cd(II) was quantified by SWV, with Cd(II) preconcentration at −0.75 V for 30 or 60 s (0.05 V/s, potential step 0.0051 V, pulse width 0.01995 V). Metal quantification was performed by duplicate, with the corresponding standard deviation calculation.

## Results

### Metal Removal by EPS and Recovery Assays

**FIGURE 3:**
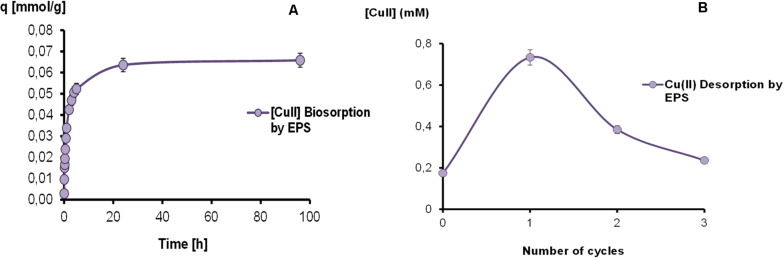
Kinetics of copper biosorption **(A)** and desorption cycles **(B)** mediated by EPS in a batch reactor.

The results obtained by Cu-EPS interaction are shown in Figure 3A. At 24 h the adsorption process almost reached the equilibrium with a metal retention of 0.064 ± 0.001 mmol g^–1^ EPS. Moreover, EPS could retain non-significant amounts of copper, registering a highest value 0.066 ± 0.001 mmol g^–1^ EPS at 96 h. These results evidenced a range of copper adsorption from 79.7 to 82.8% at 24 and 96 h, respectively.

Results of copper desorption assays on EPS-Cu can also be observed in Figure 3B, showing a metal recovery of 39.6, 20.8, and 12.8% for the first, second and third cycle, respectively. After the global HCl treatment, a total desorption of 73.2% was achieved.

**FIGURE 4:**
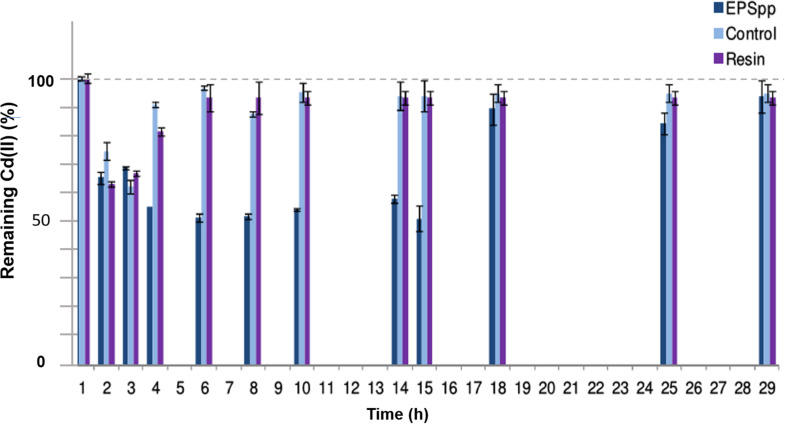
Percentage of remaining Cd(II) in time in a continuous flow bioreactor, using the EPS as biosorbent and Chelex^®^ 100 resin as reference.

In Figure 4, the results obtained for the removal of Cd(II) in time (h) in the continuous flow bioreactor are shown as % of remaining metal in solution. As can be seen in the negative control, a diffusional effect as a difference in Cd(II) concentrations was registered. Approximately shortly after 4 h of experience, the concentration was comparable to the initial one. For the system containing EPS as biosorbent, from the first half hour a decrease in the concentration of Cd(II) in the outlet effluent was detected, probably related to a diffusion process. After 1.5 h, a decrease in the concentration of the cation in solution was observed, reaching approximately a 50% of retention after 7 h, associated with the biopolymer presence. The saturation was achieved after 12 h. On the other hand, as reference, the same experiment was carried out using a commercial ion exchange resin (Chelex^®^ 100), with almost a 40% of maximal removal. Under these conditions, the results showed a better performance of the biopolymer than the synthetic resin.

After treating Cd(II)-diatomite with both *P. veronii* 2E and *P. aeruginosa* PA01 surfactants, 35.2% and 42.1% of metal desorption was, respectively, obtained. Belonging to different chemical structures both biosurfactants behaved as metal ligands recovering Cd(II) with similar yields.

### Characterization of Cu-NPs Biosynthesized by *Pseudomonas veronii* 2E

**FIGURE 5:**
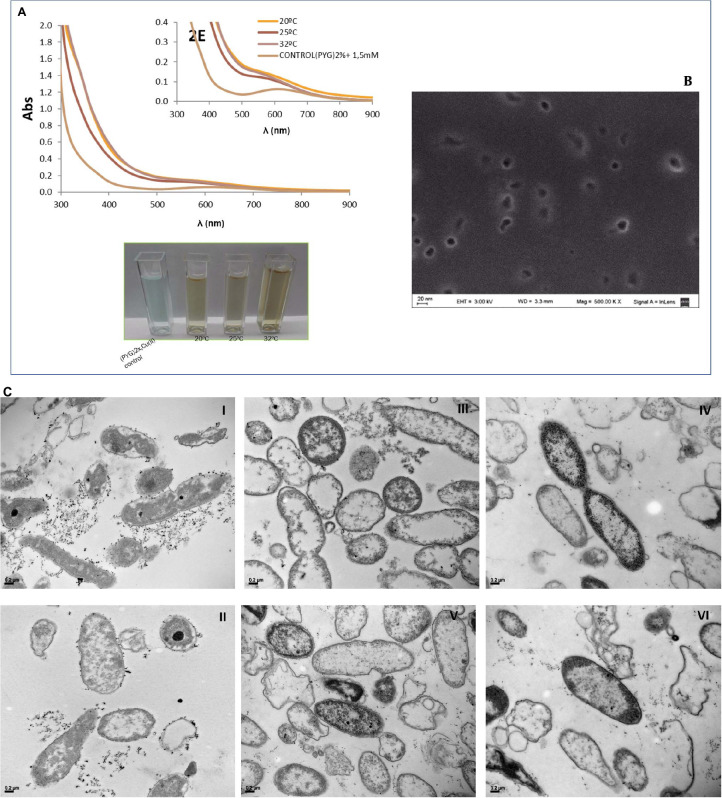
Visual color changes and absorption spectra of *P. veronii* 2E culture supernatants after 5 days-growth in presence of CuSO_4_ **(A)**. SEM Microphotographs for extracellular Cu-NPs detection and characterization from culture supernatants **(B)**. TEM Microphotographs of aggregates belonging to cell-associated Cu-NPs: images I and II correspond to Cu(II) free cultures, while images III, IV, V, and VI show cells grown in 1–1.5 mM CuSO_4_ **(C)**.

After 5 days, a visual color change from blue to reddish brown was observed on culture supernatants containing 1.0 and 1.5 mM as shown in Figure 5A with an inhibitory effect on cell growth at 2 mM of CuSO_4_, according to previous studies where Minimal Inhibitory Concentrations of Cu(II) and Cd(II) were determined (Vullo et al., 2008). This color change could be related to extracellular Cu-NPs biosynthesis consistent with the absorption spectra obtained with a 600 nm peak (Figure 5A) independently of the incubation temperature while comparing to cell-free controls. Amorphous 20 nm mean size particles were evidenced by images of SEM analysis (Figure 5B).

In addition, intracellular Cu-NPs were detected by TEM analysis as shown in Figure 5C, images III, IV, and V. Aggregates can be observed associated to cell surfaces in images belonging to Cu-treated bacteria while compared to non-treated control ones (Figure 5C, images I and II).

### Electrochemical Behavior of CPE and CPEM

**FIGURE 6:**
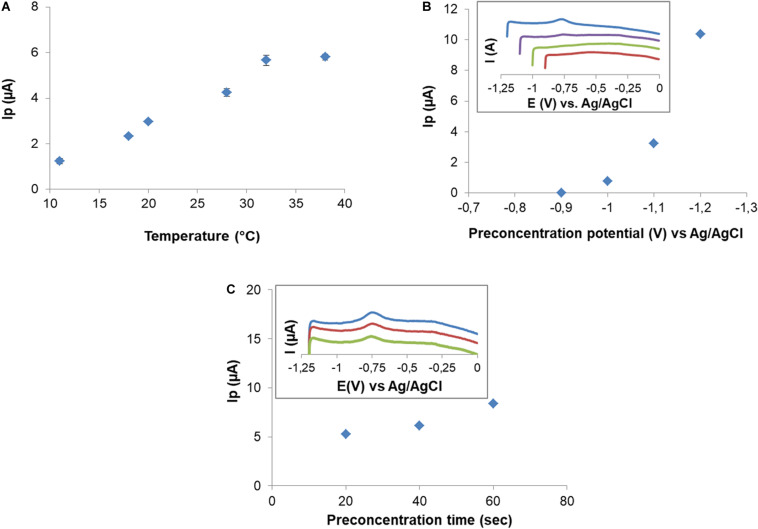
Effect of temperature on peak current after 10 μM Cd(II) exposition **(A)**. Effect of preconcentration potential on Cd(II) peak current. Insert graphic: SWV in 0.1 M NaNO_3_ for preconcentration potential –0.9 to –1.2 V **(B)**. Effect of preconcentration time on peak current. Insert graphic shows SWV for preconcentration times: 60 s (blue), 40 s (red), and 20 s (green) **(C)**.

Room temperature fluctuations clearly affected Cd(II) SWV signals. For that, a decision was taken regarding temperature control during the metal retention step. Temperature effect was studied at an initial concentration of 10 μM Cd(II). Figure 6A shows an increase in Cd(II) peak current in the range of 18–32°C followed by signal stabilization. Therefore, the following experiments were carried out at 32°C. Retention occurs with no change of the oxidation state, thus metals are retained as Cd(II) and Cu(II).

After retention, electric signal is produced by the application of a deposition potential (DP), which forces the retained Cd(II) to reduce on the electrode surface, followed by a potential scan (SWV) in the positive direction. To study the effect of DP on Cd(II) signal, potentials in the range from −0.9 to −1.2 V were applied, using a CPEM exposed to 102.5 μM. Cd(II) signal was observed from DP = −1.0 V and increased as DP became more negative (Figure 6B). Three deposition times (DT) of 20, 40, and 60 s were tested at DP = −1.2 V (Figure 6C). As expected, peak current increased as the DT increased. For the sake of sensitivity, the DP and DT were −1.2 V and 60 s for these electrochemical experiments, respectively. After each measurement a residual peak in the same position of Cd was observed. Surface cleanliness needed to be ensured, and it was also explored for electrode reuse. The residual metal signal was eliminated by immersing the electrode in MES 0.5 M pH 5.5 for 5 min. Surface regeneration was verified by SWV.

**FIGURE 7:**
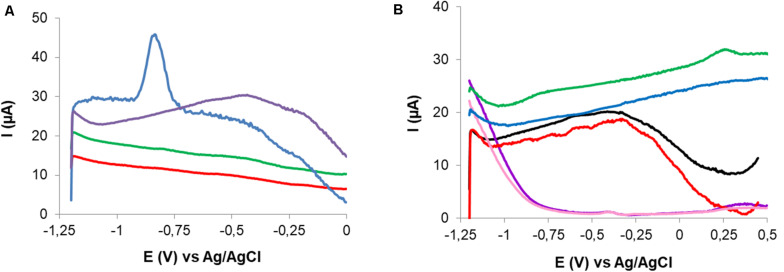
Comparison of the responses to Cd(II) exposure of CPE and CPEM in 0.1 M NaNO_3_. CPE, exposed to 102.5 μM Cd(II) (red) and baseline (green); CPEM, exposed to 50 μM Cd(II) (blue) and baseline (violet) **(A)**. Comparison of the responses to Cu(II) exposure of CPE and CPEM and effect of supporting electrolyte. CPEM exposed to 15 μM Cu(II) in 0.003 M HNO_3_ (green) and in 0.1 M NaNO_3_ (red). Baselines for CPEM in 0.1 M NaNO_3_ (black) and 0.003 M HNO_3_ (blue). CPE exposed to 100 μM Cu(II) in 0.003 M HNO_3_ (violet) and baseline (pink) **(B)**.

After cadmium exposure a characteristic peak ca −0.82 V was observed in CPEM curves. No signal was observed in the response of the non-modified electrode (CPE), which is an evidence of the retention of Cd(II) related to *P. veronii* 2E immobilized cells (Figure 7A).

Conditions for Cu(II) quantification were evaluated with CPEM after exposure to 15 μM Cu(II) for 5 min without stirring. No SWV signal was registered when exploring DP in the range of −0.5 to −1.2 V using 0.1 M NaNO_3_ as supporting electrolyte. However, a well-defined anodic peak corresponding to Cu(II) was obtained after changing the supporting electrolyte to 0.003 M HNO_3_. Cu signal appeared at 0.15 V using DP = −1.2 V and DT = 60 s. In this case the SWV signal was detected in the positive scan corresponding to Cu(0) oxidation. Additionally, no significant signal was observed for CPE exposed to Cu(II) solution in 0.003 M HNO_3_ electrolyte solution. Surface cleanliness and the possibility of electrode reuse were also explored. In fact, an anodic peak was evidenced corresponding to residual Cu(II). In this case, successful surface regeneration was achieved by immersion of the CPEM in 1.5 M HNO_3_ for 30 s (Figure 7B).

**FIGURE 8:**
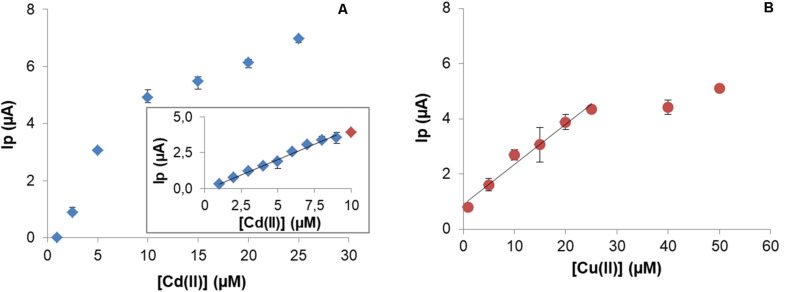
Calibration curve for Cd(II) under optimal experimental conditions. Insert graphic shows linear range between 0.6 and 9 μM (*y* = 0.4279x – 0.1121; *R*^2^ = 0.9902) **(A)**. Calibration curve for Cu(II) and linear range between 1 and 25 μM (*y* = 0.14536x – 0.7377; *R*^2^ = 0.99794) **(B)**.

After optimization of the experimental conditions, calibration curves were obtained. Cd(II) quantification on CPEM was explored between 1 and 25 μM. This microbial biosensor exhibited a linear behavior for 1–9 μM range (Figure 8A), with LOD = 0.2 μM and LOQ = 0.6 μM.

In the case of Cu(II), the analytical characterization was evaluated for concentrations in the range 1–50 μM Cu(II). A typical response was observed with a linear range up 25 μM with LOD = 2.4 μM and LOQ = 8.1 μM (Figure 8B).

## Discussion

Exopolymeric substances produced by *Pseudomonas veronii* 2E are mainly composed of carbohydrates [48% (m/m)], proteins [37% (m/m)], total phosphorus [14% (m/m)], and uronic acids [1% (m/m)] (Ferreira et al., 2017). Similar results were obtained by Ajao et al. (2020) who recovered 62–77% wt from wastewater, of which 44% corresponded to carbohydrates. This means that EPS are a complex matrix of biopolymers with carbohydrates as main components. In this work, 1.04 g EPS as dry weight was obtained from 48 h-cultures associated with the stationary phase of growth. Parmar (Parmar et al., 2020) extracted 1.34% EPS (w/v) but after 72 h at the same temperature (25°C) and concluded that increasing temperature or incubation time decreased EPS yield. Although Daniel (Daniel et al., 2016) proved that *Pseudomonas veronii* 2E evidenced an optimal biofilm development in PY broth, Ferreira (Ferreira et al., 2017) showed the highest EPS production in a minimal medium with glycerol as sole carbon source and at stationary rather than exponential phase of growth. Also, Ferreira (Ferreira et al., 2017) reported that both nitrogen and carbon sources were factors that affected EPS secretion among other parameters such as temperature and culture state. Interestingly, the maximal EPS production by *P. veronii* 2E was observed in M9 broth supplemented with glycerol at 25°C. Being glycerol a by-product of the biodiesel industry, EPS biosynthesis will contribute to the development of a sustainable biotreatment process. On the other hand, working at 25°C implies low energy requirements which directly impact on operating costs, highly desirable to an environmental bioremediation design.

Besides, the extraction method used had a significant effect on the EPS composition. In this case, using ethanol with successive centrifugations, neutral carbohydrates were mainly precipitated corresponding to the resulting biopolymeric structure. Moreover, in Ajao’s work (Ajao et al., 2020), a similar EPS recovery method was applied, with a higher uronic acids proportion obtained.

Exopolymeric substances complex matrix contains associated different functional groups such as carboxylic acid, phosphate diester / monoester groups, and amino groups involved in acid-base equilibrium being either neutral or anionic depending on pH. At a working pH (5.5) these polymers present an anionic character, an interesting property from the biotechnological point of view since they are potentially applicable in effluent treatments to remove ERM (Cd, Zn, Cr, Hg, Pb, Cu, etc.). Regarding the results obtained, EPS adsorbed 82.8% of 1 mM Cu(II) at 96 h. Also, it was proved that EPS desorbed 73.2% of the metal retained; an attractive quality when looking for metal recycling. Comparable results were obtained by Ajao et al. (2020), exposing EPS from wastewater against 50 mg L^–1^ Cu(II), retaining 99.9% and desorbing 86%. Focusing on Cd(II) retention, higher yields than those obtained with the commercial resin were achieved under the experimental conditions assayed. On the other side, Cd(II) recovery by complexation using biosurfactants as ligands resulted in an interesting desorption way from classical adsorbents as diatomite. Therefore, EPS produced by *Pseudomonas veronii* 2E exhibited properties as a suitable sorbent for copper and cadmium and a potential candidate to be applied in biotreatment processes, to both removal and recovery metals for recycling.

Bacterial cell walls are the place where binding of metal ions may occur because of the presence of different functional groups such as carboxylate, hydroxyl, phosphate, sulfate, and amine. This natural affinity established between metal ions and groups present in bacterial cell walls can be used in the retention of metals. Composition of whole cell walls of *P. veronii* 2E has been studied by FTIR (Ferreira et al., 2020). Briefly, peaks in the spectra were assigned to amino, phosphate, amido, sulfhydryl, carboxyl, and hydroxyls present in polysaccharides, protein, nucleic acids, and lipopolysaccharides. The shape and position was clearly affected by the exposure to Cu(II), Cd(II), and Zn(II), which revealed their significant role in the chelation of these metal ions. Also in agreement with these results, a previous study regarding metal biosorption by *P. veronii* 2E (Vullo et al., 2008) provided the optimized conditions for Cu(II) and Cd(II) retention which were used in this work: pH 7.5 (10 mM HEPES) for Cd(II) and pH 5.5 (10 mM MES) for Cu(II). These were crucial for establishing the initial working conditions for the CPEM, since the pH of the preconcentration solution affects not only the solubility of the metal ions but more importantly, modifies the ionization state of the functional groups (carboxylate, phosphate, and amino groups) located in the cell wall. At the selected pH carboxylate and phosphate are negatively charged and provide a possible site for the biosorption of metal ions.

As far as known there are no reports of the modification of CPE with *Pseudomonas* for Cd(II) and Cu(II) detection. CPE modified with *P. veronii* 2E allowed metal retention and subsequent detection. Peaks observed at −0.82 V and at 0.15 V from CPEM corresponded to Cd(II) and Cu(II) reduction, respectively, because of the nature of the potential sequences applied (Figure 7). Since the signal is generated by the electron transfer on this particular CPEM surface, any potential shift from the standard value could be explained not only by the surface used but also by strong metal-*P. veronii* 2E binding (Martínez-Sánchez et al., 2012). Since no peaks were observed at those potentials on the CPE surface, the occurrence of electric signals was derived from *P. veronii* 2E-metal biosorption onto the bacterial-modified electrode surface.

Cd(II) retention required working at controlled temperature (32°C) indicating a dependence between metal-biosorption and temperature, which depend on the nature of the system. In fact, an increase in retention with increasing temperature suggests an endothermic nature of the biosorption reaction (Daneshfozoun et al., 2017).

Preconcentration potential was studied, and it was observed that from potential higher than −0.9 V peak corresponding to Cd(II) was observed and it increased gradually as potential was more negative (Figure 6). For that reason, −1.2 V was selected as preconcentration potential where metal biosorbed is reduced and then oxidized by scanning potential in the positive direction in SWV.

Regeneration of the electrode surface is a very important issue. A step between experiments was required for surface regeneration both for Cd(II) and Cu(II), due to the persistence of a residual signal. It involved the use of acidic media to release the biosorbed metal from the electrode surface. Surface regeneration was easy to achieve. The use of this solution is required to restore the ability of the aforementioned functional groups to interact with metal ions. This acidic treatment is also used in the case of the determination of Pb(II) using a carbon paste electrode modified by treated-*Pennisetum setosum* (Ouangpipat et al., 2003). However, different treatments have been proposed for this purpose regarding modified carbon paste electrodes, which comprises simple procedures like polishing the electrode on A4 paper 80 g m^–2^ (Takeuchi et al., 2007), squeezing out a small amount of the carbon paste (Devnani and Satsangee, 2015) or immersion in stirred deionized water (Özcan et al., 2018).

Typical responses have been obtained with both Cd(II) and Cu(II) quantification. In general, the LOD and LOQ values are close to reported values for other similar constructions. When used for Cd(II), linearity was achieved in the range 0.6–9 μM with LOD 0.2 μM. Compared to other works, CPEM has a LOD of the same magnitude for other modifications in the literature, for example a CPE modified with *Eichhornia crassipes* has an LOD = 0.04 μM (Jackfama et al., 2019) or with coconut, with a LOD = 0.93 μM (Rajawat et al., 2014).

In the case of Cu(II), an LOD = 0.2 μM and a linear behavior from 0.6 to 25 μM was obtained. In order to compare to similar constructions, Alpat reported on a CPE modified with 5% *Circinella* sp. (Alpat et al., 2008) with an LOD = 0.054 μM. Considering that the authors exposed the modified electrode during 30 min for Cu(II) retention, which is six times the retention time used with the CPE modified with *P. veronii* 2E, the performances of both biosensors are quite similar both in sensitivity and LOD. In fact, from an experimental point of view, faster tests are a good solution when dealing with a high number of samples.

Regarding biosynthesis of Cu-NPs, both extracellular and cell-associated aggregates were detected by both spectrophotometric and SEM and TEM techniques. Extracellular Cu-NPs presence could be confirmed by a distinct reddish brown color solution after cell growth in a CuSO_4_ supplemented culture broth. An absorption peak which increased with Cu(II) concentration was registered at 600 nm, consistent with other reported biogenic Cu-NPs. In literature, a wide range of absorption wavelengths within UV-visible spectrum were reported, depending on obtaining methods, originating copper salts and coating compounds. Therefore, Cu-NPs synthesis conditions contributed to their stabilization and played relevant roles in final solution properties and spectrophotometric responses and could be differentiated from CuO-NPs (Ammar et al., 2019; Thiruvengadam et al., 2019; Noman et al., 2020; Siddiqi and Husen, 2020). In addition, SEM images revealed approximately 20 nm-size extracellular NPs, coincident with other biogenic Cu-NPs from different sources (Ghosh, 2018; Siddiqi and Husen, 2020). Focusing cell-associated Cu-NPs, aggregates were visualized in TEM images while comparing to non Cu-treated cultures. Since no reports of intracellular Cu-NPs were found in literature (Ghosh, 2018), further studies should be carried out applying TEM-EDS and FTIR techniques to facilitate an elemental analysis and thus to elucidate the structure of these aggregates.

Responses of *P. veronii* 2E and its products – biosurfactants, bound and soluble EPS- allowed Cu(II) and Cd(II) removal, recovery and biosensing resulting in a multiple and versatile tool for sustainable wastewater biotreatments.

## Data Availability Statement

The original contributions presented in the study are included in the article/supplementary material, further inquiries can be directed to the corresponding author.

## Author Contributions

MB work was focused on Cu(II) removal and recovery from EPS. IL studied the development of Cu and Cd biosensors. MF worked in Cd retention by EPS and biosynthesis of Cu-NPs. SR and DV conceived the study and designed the experimental setup. RC collaborated with Cu-NPs manipulation and TEM. All authors were involved with manuscript ideas and editing of relevant sections.

## Conflict of Interest

The authors declare that the research was conducted in the absence of any commercial or financial relationships that could be construed as a potential conflict of interest.
